# The role of religious leaders on the use of HIV/AIDS prevention strategies among young people (15–24) in Lira district, Uganda

**DOI:** 10.1371/journal.pone.0276801

**Published:** 2022-10-27

**Authors:** Tom Murungi, Irene Kunihira, Pamela Oyella, Moses Mugerwa, Peruth Gift, Mercy Jane Aceng, Lydia Abolo, Sean Steven Puleh

**Affiliations:** 1 Department of Midwifery and Nursing, Faculty of Health Sciences, Lira University, Lira City, Uganda; 2 Department of Epidemiology and Biostatistics, Faculty of Public Health, Lira University, Lira City, Uganda; 3 Department of Mental Health, Faculty of Health Sciences, Lira University, Lira City, Uganda; Ohio University, UNITED STATES

## Abstract

**Background:**

Young people (15–24 years) bear the highest burden of new infections and are particularly vulnerable because of their highly risky behavior such as early sexual activity. There is paucity of information on the role of religious leaders in the multi-sectoral fight against HIV/AIDS. We examined the role of religious leaders in the use of HIV prevention strategies among young people.

**Methods:**

A cross sectional study was conducted between March and April 2021 among 422 randomly selected young people in Lira district, Uganda. An interviewer administered a questionnaire to the young people in order to collect quantitative data. A total 20 key informants were purposively sampled and interviews were conducted with religious leaders using a key informant’s interview guide. Data was collected on social demographics, HIV prevention messages, and awareness about HIV prevention strategies. Data was analyzed using Stata version 15 using proportions, means, percentages, frequencies, and logistic regression analysis at a 95% level of significance. Qualitative data was analyzed using thematic content analysis and the major themes were generated from the participants’ responses.

**Results:**

About 57.1% (241/422) of the respondents were females. The prevalence of use of HIV prevention strategies among young people was 69.4%. Factors significantly associated with the use of HIV prevention included completing the primary level (aOR 4.95, p< 0.05), completing at least A level (aOR 8.85, p < <0.05), Awareness of HIV prevention strategies advocated for by religious leaders (aOR 0.02, p<0.001), religious leaders provided targeted HIV prevention messages (aOR 2.53, p<0.01), Advocacy for abstinence outside marriage and fidelity in marriage (aOR 35.6, p<0.01), Religious leaders preaching about HIV prevention (aOR 4.88, p<0.001). Qualitative data indicated that a section of religious leaders recommended abstinence/faithfulness. Condom use was the most discouraged HIV prevention strategy. However, most religious leaders agree with the fact that they have a role to play in HIV prevention, which includes sensitization, teaching and organizing sermons about HIV prevention.

**Conclusion:**

The use of HIV prevention strategies advocated for by religious leaders among young people was nearly 70%. This finding indicates that religious leaders have a role to play in HIV/AIDS prevention among young people in the Lira district. This calls for the involvement of religious leaders in HIV prevention programs tailored to prevent new infections of HIV among young people.

## Introduction

Globally, 38 million people live with HIV, 1.5 million people become infected yearly and about 34.7 million people have died from AIDS related illnesses since the start of the epidemic [1]. An estimated 40 percent of the new HIV infections worldwide occur among young people aged 15–24 years [2]. In addition, over 30% of all daily new infections are projected to occur among young people aged 15–24 years, with males and females disproportionately affected at 12% and 30% respectively [3]. Data from UNICEF shows that 460,000 young people (10–24) were newly infected with HIV [4]. Sub-Saharan Africa (SSA) bears the greatest burden of HIV/AIDS, accounting for the greatest number of new infections and deaths worldwide [5]. Young people account for approximately 20.6% of Uganda’s population [6] with a HIV a prevalence of 1.8% [7].

Young people (15–24 years) are particularly important because they bear the highest burden of HIV new infections [1]. In addition, young people are regarded as vulnerable because they tend to engage in the riskiest behaviors, such as engaging in sexual activity, amidst several challenges, including physiological changes, peer pressure, among others [8]. In this study, risky behaviors refer to behaviors characterized by different hazardous actions such as premarital sex, multiple sexual partners, and unprotected sex. In Uganda, HIV prevention interventions are aligned with the drivers of the epidemic. These include; delay sexual debut, elimination of unsafe sex, promoting correct and consistent condom use in the general population and high risk groups, reducing multiple, especially concurrent sexual partnerships, discourage cross-generational and transactional sex, wide coverage of safe male circumcision, reducing community viral load through anti-retroviral therapy and appropriate ARV prophylaxis [9, 10]. However, there is limited data on how effective these strategies have been especially among the young people. Some studies have reported early sexual debuts among students, which is a risk factor for sexually transmitted infections and HIV acquisition [11]. Uptake of HIV prevention services among young people is low and there is no gold standard intervention in regards to HIV prevention among young people in SSA [12, 13]. HIV prevention strategies for young people necessitate multifaceted interventions [14]. The national HIV prevention strategy recommends the use of all institutions including religious institutions to deliver HIV prevention messages and advocacy services to the general populations [10]. Previous research has suggested linkages between the involvements of Faith-Based Organizations (FBO) in campaigns to prevent HIV/AIDS [15, 16]. FBOs spend a large proportion of their time engaging with the community, thereby shaping social norms, attitudes, beliefs and people’s reality with regard to sexual self-understanding, making them crucial partners in HIV/AIDS prevention [17]. Some studies have reported mixed information about religious organizations at an institutional and individual level. The Catholic church for example has discouraged condom use and used stigmatizing language while at the same time religious leaders have been regarded as supportive [16]. Which makes the role of religious leaders unclear as an effective tool that can be used in HIV prevention among young people. For example, there is no evidence of studies that have evaluated to what extent religious leaders have played a role in HIV/AIDS prevention programme among young people as fundamental actors. Religious leaders in this study are individuals recognized as having authority within a specific religion, such as Pastors, Reverends, Bishops, Catechists, Priests, Sheikhs, and Imams. Uganda is a multi-denominational country, with approximately 82% of the population being Christians, 14% being Muslims, and less than 5% belonging to other religions, according to the 2014 national census [18]. Given the status religious leaders hold in many societies, they frequently have contact with communities and can utilize their pulpits to reach out to many, especially young people in hard-to-reach areas [19]. In addition, religious leaders can use their positions to break the silence surrounding HIV/AIDS, shape social values, disseminate accurate information and influence opinion [20]. However, the levels and intensity of engagement by religious leaders and how they are likely to influence the use of HIV prevention strategies among young people has not been studied.

HIV prevention strategies refer to ways in which individuals can reduce the risk of HIV infection by limiting exposure to risk factors. Condom use, safe male circumcision, pre-exposure prophylaxis (PrEP), elimination of mother-to-child transmission (EMTCT), testing and counseling for HIV and sexually transmitted infections (STI) have been adopted as some of the HIV prevention strategies. However, there is a paucity of information regarding how religious leaders can play a role in preventing HIV among young people. Evidence from a similar setting focused on religious leaders’ involvement in HIV prevention and care for gays, bisexual men, and others who have sex with men [21]. Some studies have only looked at involving religious leaders in HIV care and treatment at health facilities [22]. Because of the importance of religious beliefs in the prevention of HIV/AIDS, it is critical to assess religious leaders’ roles in the use of HIV prevention strategies among young people in Lira district. This study has generated evidence-based data for programming to improve the involvement of religious leaders in the use of HIV prevention strategies among young people.

## Methods

### Study design

A cross-sectional survey using quantitative and qualitative methods was carried out in March and April, 2021.

### Study setting

The study was conducted in the greater Lira district, which is divided into Lira City (urban) and Lira District Local Government (regarded as rural), in Northern Uganda. Lira City is further divided into two (02) divisions Lira City East and Lira City West, and Lira District Local Government divided into Erute North and Erute South. Lira district is bordered by Pader district to the North, Otuke to the East, Dokolo to the South West and Kole to the West. It has an estimated population of about 478,500. It is about 380km from Kampala, the capital of Uganda.

### Study population

The study was conducted among 422 young people aged between 15 to 24 years living in Lira district, Northern Uganda, to collect quantitative data. The term young people was used to represent a period of changeover from childhood and dependence to adulthood and independence, a stage thought to have several behavioral, social challenges and vulnerability [23]. We also conducted 20 key informant interviews with religious leaders who are recognized as having authority within their respective religions, such as pastors, reverends, deacons, bishops, catechists, priests, sheikhs, and imams.

### Sample size estimation

The Cochran’s formula (1997) was used to calculate the sample size for young people.

Based on the assumptions that proportion of those who used the HIV prevention strategies advocated for by the religious leaders was 50% (p = 0.5), proportion who did not use at 50% (q = 1-p), and margin of error (d = 0.05, and d = margin of error, our sample size was = 384. With a 10% non-response rate, our sample size was n = 422. Key informant interviews were conducted with 20 religious leaders who were sampled purposively.

### Data collection procedure

This was a mixed method study using both quantitative and qualitative methods. We collected qualitative data first, followed by quantitative data. Quantitative data was collected using interviewer administered questionnaires and an interview guide was used to gather information from the religious leaders of the different denominations under the study. We developed our study tools and adapted some interview questions from other studies done in a similar setting [24, 25]. Our study tools were pretested on 20 young people and adjusted accordingly.

Written consent was sought from all participants before screening to determine their eligibility to participate in the survey. Assent and consent was sought from all respondents who were 15 to 17 and those at least 18 years respectively before their participation. Participants were interviewed in turn until the quota was reached per village. These interviews took about 15–20 minutes on average. Consenting religious leaders were interviewed using an interview guide to collect qualitative data and the interviews lasted between 20 and 25 minutes. All research assistants were trained prior to data collection. All study tools were reviewed for completion at the end of data collection to ensure the quality of the data.

### Sampling criteria

The religious leaders and young people were chosen using a multi-stage cluster sampling technique. One county/division was selected from each cluster using simple random sampling. Three sub-counties were sampled from each county using a table of random numbers. Three (3) parishes/wards were sampled from each sub county using a table of random numbers. A simple random sampling technique was used to select one village/ cell from each parish/ward. Convenience sampling was used to select the young people and religious leaders to include all dominant religious denominations. Those who met the inclusion criteria and consented were included. Those who were eligible were recruited from the villages/cells until the sample size was reached.

### Data management

In the field, questionnaires were checked for completeness. For participants who were unable to read or write, a witness was involved to ensure transparency. Data was entered into SPSS version 23 with consistency checks to ensure correctness. The dataset was cleaned for out-of-range values and exported to STATA 15 (StataCorp, College Station, TX) software.

For the key informant interviews, all the interview recordings were checked at the end of every interview to ensure completeness. All questions were asked in order and, where necessary, probing was done to make the questions clearer for the respondent. Audios were kept on hard drives, only accessible to the study team. Unique identification numbers were used to identify participants to ensure confidentiality.

### Data analysis plan

We summarized and conducted a descriptive analysis to determine the proportions of the different variables in the respondents’ characteristics. In this study, categorical variables such as place of residence and sex refer to biological differences between females and males, such as chromosomes, sex organs, and endogenous hormonal profiles. Marital status and level of education were analyzed and expressed as frequencies and proportions, whereas continuous variables such as age were expressed as means (standard deviation). At the univariate level, Chi-square tests were performed to determine the association between dependent and independent variables. Bivariate analyses were used to compute the unadjusted associations between the use of HIV prevention strategies and independent variables, including socio-demographic characteristics (such as age, marital status, level of education, sex and occupation of participants), and HIV prevention strategies advocated for by religious leaders (such as; condom use, being faithful, abstinence, raising awareness, advocating for voluntary medical male circumcision). The results were expressed in terms of odds ratio at a 95% level of confidence and a P value < 0.05. Variables that were significant in the bivariate analysis (p < 0.05) were considered for the multivariable analysis. We conducted multivariable analysis using the independent variables that are statistically significant during the bivariate analysis and those considered plausible although not significant against the dependent variable. We assessed for extraneous variables before building the final model. Logistic regression was performed to come up with a suitable model to explain the predictors of use of HIV prevention strategies among young people and the statistical significance of p<0.05. Qualitative data was analyzed using thematic content analysis according to [26] that describes seven steps of data analysis including1) transcription, 2) reading and familiarization 3) coding, 4) searching for themes, 5) review of themes, 6) naming the themes, 7) finalizing the analysis and interpretation of the results.

### Ethical consideration

Approvals were sought from Gulu University Research and Ethics Committee (GUREC-2021-34) and Resident District Commissioner to conduct the study in Lira district. Permission to conduct the study was further obtained from the heads of different churches and mosques before data collection. Written informed consent was sought from all respondents before interviews were conducted. Refreshments were provided to the respondents at the end of the interview. Where minors were involved, we obtained consent from the parents before being involved in the study. The informed consent process included providing explanations about the purpose and objectives of the study, the procedures, benefits and risks to be borne by the respondent and reassurance of confidentiality. To ensure confidentiality, all information regarding respondents remained confidential within the study team. Unique numerical identifiers were used for computer-based data entry and to conceal the identity of the participants. All respondents were free to participate without any form of coercion.

## Results

### The prevalence of use of HIV prevention among young people

The prevalence of the participants who use (binary outcome) HIV prevention strategies advocated for by the religious leaders was 69.4% (293/422).

### Social demographic characteristics of the respondents

A total of 422 participants were interviewed using an interviewer administered questionnaire with a 100% response rate. The mean age of the respondents was 19.18 (SD = ±2.6) with 56.9% (240/422) in the age category of less than 20 years. About 71.8% (303/422) of the respondents were single and 57.1% (241/422) were female. The majority of respondents, 45% (190/422), had completed an ordinary level of education (Table 1).

**Table 1:** Shows the young peoples’ socio-demographic characteristics.

Variables	Frequency (n = 422)	Percentage (%)	P -value
**Sex of respondents**			
Female	241	57.1	0.721
Male	181	42.9	
**Age of respondents**			
<20	240	56.9	
≥20	182	43.1	0.023
**Place of residence of respondents**			
Rural	202	47.9	
Urban	220	52.1	0.874
**Religion of respondents**			
Catholic	138	32.7	0.896
Protestant	112	26.5	
Pentecostal	60	14.2	
Seventh day Adventist	85	20.1	
Orthodox	11	2.6	
Muslim	16	3.8	
**Marital status of respondents**			
Single	303	71.8	0.215
Cohabiting	22	5.2	
Never married	50	11.9	
Separated	13	3.1	
Married	34	8.1	
**Highest education level of respondents**			
No formal education	20	4.8	<0.001
Completed Primary education	144	34.1	
Completed O’level education	190	45.0	
Advanced level plus	68	16.1	
**Occupation of respondents**			
Non formal	62	14.7	0.516
Student	291	69.0	
Business	49	11.6	
Formal employment	20	4.7	

### HIV prevention strategies provided by religious leaders

The majority of participants, 85% (362/422), reported that their religious leaders advocate for abstinence. In addition, the majority, 95% (401/422) of the participants, reported that their religious leaders advocated for faithfulness (Table 2). Participants in the qualitative interviews strongly supported or advocated for abstinence, particularly among unmarried young people, and for being faithful to one partner among married people. One respondent reported that:

**Table 2:** Perceptions and HIV prevention strategies provided by religious leaders.

Variables	Frequency (n = 422)	Percentage (%)	P-value
Do your religious leaders advocate for abstinence outside marriage?			
No	60	14.2	
Yes	362	85.8	<0.001
Do your religious leaders advocate for fidelity in marriage?			
No	21	5.0	
Yes	401	95.0	<0.001
Do your religious leaders advocate for Condom use?			
No	303	71.8	
Yes	119	28.2	0.577
Do your religious leaders advocate for voluntary medical male circumcision?			
No	216	51.2	
Yes	206	48.8	<0.003
Do your religious leaders advocate for HIV Counseling and Testing?			
No	42	10.0	
Yes	380	90.0	<0.001
Do your religious leaders preach about HIV prevention?			
No	147	34.8	
Yes	275	65.2	<0.001
Are there specific HIV prevention strategies provided at your church/mosque?			
No	220	52.1	
Yes	202	47.9	<0.001
Reported religious leaders involved in HIV awareness campaign among young people			
No	161	38.1	
Yes	261	61.9	<0.001
Do you get routine HIV counseling at your church/mosque?			
No	222	52.6	
Yes	200	47.4	<0.001
The religious leaders have a strong influence on the choice of HIV prevention strategies			
Strongly disagree	34	8.1	<0.001
Disagree	72	17.1	
Agree	200	47.4	
Strongly agree	116	27.4	
The preaching of your religious leaders affects choice of HIV prevention strategy			
Strongly disagree	47	11.1	<0.001
Disagree	78	18.5	
Agree	202	47.9	
Strongly agree	95	22.5	
The involvement of religious leaders in HIV/AIDS prevention helps prevent HIV among young people			
Strongly disagree	39	9.2	<0.001
Disagree	78	18.5	
Agree	188	44.6	
Strongly agree	117	27.7

*"Abstinence is the best*. *We discourage premarital sex for two reasons*: *one*, *you don’t go when there isn’t time*, *and there is time for everything*. *Sex is good*, *but in a marriage setting*, *it’s inappropriate*. *"So*, *according to Galatians 5*:*19*, *we reject and condemn fornication among young people*, *and we discourage and condemn adultery*. *“The only good way is abstinence and patience*. *"*Male, SDA, R16, 38 years, March 2021.

As far as condom use is concerned, religious leaders have varying opinions concerning their support for condom use. Therefore, the message regarding condom use as one of the HIV prevention strategies by religious leaders cannot be ignored. A significant proportion, 71.8% (303/422) of the respondents, reported that their religious leaders discouraged condom use (Table 2). This result was further supported by a section of religious leaders who reported discouraging condom use among young people. Those who don’t support condom use argue that it promotes fornication and adultery among the youth. Some of the key informants remarked:

*"Using condoms is not good for them because it allows them to engage in sexual relationships*, *which can easily lead to the spread of HIV/AIDS among young people” When you read Exodus 20 in the Bible*, *it talks about avoiding fornication*, *and the church teaches the youth not to engage in sexual relationships”*. Male, SDA, R12, 50 years, March 2021.

According to another key informant:

*"*... *Because of respecting the law*, *just like I swore*, *it makes me accept what the church has accepted*. *But what the church has refused*, *I will also refuse"* Male, Catholic, R5, 30 years, March 2021.

A level of ambivalence was articulated by one of the key informants who was able to detach himself from his religion and reported he would encourage young people to use condoms because it would prevent them from contracting the virus, as quoted below:

*"*.... *"Leaving aside the issue of being a catechist*, *it is to advise a child to go to the hospital and get a condom in order to prevent a child from contracting the virus"* Male, Catholic, R6, 42 years, March 2021.

About 51.2% (216/422) reported that their religious leaders have never advocated for voluntary medical male circumcision (Table 2). According to some qualitative interview participants, male circumcision is not a safer method of HIV prevention among young people, as one can still contract the disease and it encourages sexual relationships. One religious leader said:

*"It makes one have uncontrollable sexual behavior*.*"* Male, Anglican, R11, 46 years, March 2021.

A significant proportion of participants, 65.2% (275/422), reported that their religious leaders preach about HIV prevention. The same message was echoed by participants in the key informant’s interviews. One participant reported:

"*We preach to them and we encourage them to carry out tests to know their status as well*. *For those that already know their status*, *we always give them words of hope that that’s not the end of life"* Male, Anglican, R19, 39 years.

About 61.9% (261/422) of the respondents reported that their religious leaders are involved in awareness creation programs among young people (Table 2). A section of the religious leaders reported not having posters/charts with HIV prevention messages in their various places of worship. If they are available, some people prefer to hang them in their places of worship. One participant was quoted as saying;

*"*...*So my opinion is that it should not be hung against the wall in the church because it’s the house of the Lord*, *but it can be hung outside the church in the compound*.*"* Female, Pentecostal, R4, 50 years, March 2021.

The majority, 47.4% (200/422) of young people, reported that religious leaders have a strong influence on their use of HIV prevention strategies. A significant number, 47.9% (202/422) reported that religious leaders’ preaching influences their choice of HIV prevention strategy. Moreover, most 61.9% (261/422) of the participants reported that their religious leaders are involved in HIV/AIDS awareness campaigns (Table 2). This result is consistent with the results of the key informant’s interviews. One participant reported:

*They have training most of the time during camp meetings*, *in the 13th week*,........... *and the church also organizes youth meetings once a month*. *We also have an Adventist Youth group that we teach about the dangers and prevention of HIV/AIDS*, *and we have books like ’Free from Addiction’ that we give people to read about HIV/AIDS*.*" All of these programs are aimed at youths as well*. *"*Male, SDA, R12, 50 years.

All the religious leaders interviewed had similar views on safe male circumcision as an HIV prevention strategy, except one respondent who had a different view. In his words he noted that;

*“One thing that we as the church do not encourage is circumcision because it makes one to have an uncontrollable sexual behavior*.*” Male, Anglican, R11, 46 years, March 2021*.

### Bivariate and multivariable logistic regression of factors associated with use of HIV prevention strategies among young people

Factors significantly associated with the use of HIV prevention include completion of the primary level (aOR 4.95, p< 0.05), completion of at least A level (aOR 8.85, p<0.05), awareness of HIV prevention strategies by religious leaders (aOR 0.02, p<0.001), provision of HIV prevention messages from religious leaders (aOR 2.53, p<0.01), Advocacy for being faithful (aOR 35.6, p<0.01), Religious leaders preaching about HIV prevention (aOR 4.88, p<0.001) (Table 3).

**Table 3:** Bivariate and multivariable logistic regression of factors associated with use of HIV prevention strategies among young people.

Variables	Use of HIV prevention strategies				
	Yes	No	Crude OR (95%CI)		Adjusted OR 95% CI
Highest level of education completed					
Education level Primary	91	53	1.72(0.671–4.394)^*NS*^	4.95(1.421–17.220)*	
Completed O’ Level	129	61	2.11(0.836–5.349)^*NS*^	2.24(0.721–6.973)	
Completed at least A’ level	63	5	12.60(3.560–44.596)***	8.85(2.018–38.794)*	
Aware of HIV prevention strategies by religious leaders	6	82	0.01(0.005–0.029)***	0.02(0.006–0.052)***	
Received HIV prevention strategies from Religious leaders	269	24	3.72(1.844–7.525)***	2.53(1.045–6.110)**	
Do religious leaders advocate for being faithful	292	109	2.17(1.315–3.575)**	35.6(3.639–348.998)**	
Do your religious leaders preach about HIV prevention	235	40	5.13(2.052–12.813) ***	4.88(2.504–9.517)***	

Level of significance

*p<0.05

**p<0.01

***p<0.001 NS Not significant

## Discussion

In our study in which we assessed the utilization of HIV prevention strategies provided by religious leaders among young people, we found nearly 70% of the participants reported using these HIV prevention strategies. In these settings, religious leaders are held in high esteem and they are viewed as trustworthy sources of information and guidance and compliance with their teaching could help prevent HIV among young people. This finding is consistent with other evidence where it is believed that religious leaders teach values whose obedience is likely to protect communities against HIV/AIDS [27]. In addition, evidence from elsewhere shows that religious leaders can help in the dissemination of health information, more specifically HIV prevention, to the communities that they serve [25, 28]. In other settings, however, some religious leaders have been reprimanded by their religious authorities and censored for preaching to their congregants on the subject of HIV/AIDS [29]. These actions are often driven by the negative association between HIV/AIDS and immorality, particularly in the form of promiscuity, and could impede progress in the fight against HIV. Furthermore, religious leaders have been accused of spreading false information; for example, making claims that prayers can heal HIV [28, 30] and unhealthy attitudes and behaviors create a barrier to prevention success among young people [31]. Among some religious leaders, HIV/AIDS is considered God’s punishment for sins such as being sexually promiscuous [32]. This implies that some religious leaders simply do not have the requisite knowledge of HIV/AIDS and its prevention.

The religious leaders’ creation of awareness was one of the reasons for the high utilization of HIV prevention strategies among young people. Our results also show that about 61.9% of young people reported that their religious leaders are involved in HIV/AIDS awareness campaigns. Our results also show that about 61.9% of the young people reported that their religious leaders are involved in HIV/AIDS awareness campaigns. This could be attributed to the fact that some religions are actively involved in HIV prevention, care and management [33]. Furthermore, some religious leaders stated that they organize workshops and seminars to teach young people about HIV prevention. This finding is in line with evidence from Senegal, where religious leaders were more likely to report teaching prevention [25]. Awareness created through religious engagements is likely to impact positively on the fight against HIV/AIDS among young people. In addition, those who were aware of HIV prevention strategies were more likely to use HIV prevention strategies as opposed to those who were not. These findings are consistent with data from other settings [34].

Our results also indicate that young people who were preached to about HIV prevention were more likely to use HIV prevention strategies provided by religious leaders. Preaching would lead to awareness creation, which empowers young people to be able to make better decisions as far as HIV prevention is concerned. Religious leaders are considered as moral authorities in their settings, and can be given platforms during religious gatherings, funerals, marriages and ceremonies, to preach about appropriate sexual behavior and other significant information regarding HIV prevention [35, 36]. Moreover, this finding is consistent with evidence from Malawi, where preaching about AIDS is a common activity for HIV prevention [21, 33, 37].

In our study, abstinence outside marriage and being faithful among the married were the most advocated strategies by religious leaders. Younger people whose religious leaders preached about abstinence and fidelity were 35.6 times more likely to use HIV prevention strategies compared to those who were not. Preaching is likely to result in more religiously enhanced behaviors that are beneficial to HIV prevention and lead to a constructive reduction in HIV spread and lower HIV prevalence [32, 38–40]. This is because abstinence and fidelity are rooted in most religious beliefs, and such values have the potential to lower the risk of HIV acquisition. The Holy Bible, for example, forbids adultery and fornication (Galatians 5:19). Our findings are consistent with what other scholars have observed elsewhere where sexual abstinence and mutual fidelity are encouraged as opposed to condom use that is viewed as undermining morality [28, 34]. This suggests that religious leaders place a greater emphasis on behavioral mechanisms, which are an important part of enforcing social values.

Our findings indicate that only 28% of young people reported their religious leaders advocating for condom use. Additionally, a huge proportion of religious leaders are not in favor of condoms being used as a prevention strategy. Condom use is viewed as a promotion of sexual immorality in many religious faiths, and in most cases, faith-based organizations have been reported to demonize condom use as promoting sinful behavior [35]. Catholicism, for example, forbids the use of condoms, even as a method of family planning. This finding is also consistent with evidence observed by other scholars in other settings [24, 25, 37, 41]. Nevertheless, some of the religious leaders in this study were able to detach themselves from their religions. For example, a section of religious leaders reported that they would advise young people to use condoms even though it is against the beliefs of their religion. As one respondent said, "many ways of HIV AIDS prevention are there, like condom use, but the church does not encourage this.

Religious leaders have shown a willingness to learn about and participate in HIV prevention in some settings, proving to be a valuable partner in the fight against the pandemic [21, 31, 34, 37, 42]. Over the years, efforts have been made to involve religious leaders in addressing issues related to HIV/AIDs. For example, an inter-religious project that provides training for religious leaders with scientific information supported by both Muslim and Christian faith teachings was piloted in 1995 and has evolved to address several HIV-related community needs [32]. However, according to some religious leaders in our qualitative results, their role was limited by a lack of training, inadequate equipment and facilitation to carry out HIV prevention programs. Similar results were obtained from a study in Nigeria [29].

However, our study had some limitations. Our data did not include the Traditional African Religious (TAR) leaders. The focus of our study was on modern religious leaders because it was challenging to detach the TAR leaders from their traditional beliefs from their spiritual components. Qualitative data collection relied heavily on KIIs that are prone to recall and respondent bias. In addition, we were unable to assess the age of initiation into sex, which would have been a good indicator of the level of risk. We did not assess the effect of other prevention strategies, for example, the media, and peer education. These are potential areas of future research in a similar setting. This study, on the other hand, was able to separate religious leaders from their religious affiliations, at least in some cases. And it highlights the importance of religious leaders and their readiness to participate in HIV prevention among young people

## Conclusion and recommendations

We have found that religious leaders can play a pivotal role in HIV prevention among young people. The predictors of use of HIV prevention strategies provided by religious leaders include completion of an advanced level of education, awareness of HIV prevention strategies by religious leaders, provision of HIV prevention messages from religious leaders, advocacy for being faithful and religious leaders preaching about HIV prevention. Our study resonates with previous findings and builds on existing literature to highlight the role of religious leaders in the use of HIV prevention strategies among young people in resource-constrained settings. The benefit of the role of religious leaders is that they are considered as moral authorities in their settings, and are given platforms during religious gatherings, funerals, marriages and ceremonies, to educate them about appropriate sexual behavior and other significant information regarding HIV prevention. Efforts to train and harmonize key messages on HIV prevention messages, improved access to information, education, and communication (IEC) materials, and improvement in the level of education of young people will promote the use of HIV prevention strategies among young people.

## Supporting information

S1 Dataset (DTA)
